# 2,3-Dibromo-1-[4-(2,3-dibromo-4,5-di­meth­oxy­benz­yl)-2,5-dimeth­oxy­benz­yl]-4,5-dimeth­oxy­benzene

**DOI:** 10.1107/S1600536810043758

**Published:** 2010-10-31

**Authors:** Ertan Şahin, Halis T. Balaydın, Süleyman Göksu, Abdullah Menzek

**Affiliations:** a Atatürk University, Department of Chemistry, 25240 Erzurum, Turkey; b Artvin Çoruh University, Education Faculty, 08100 Artvin, Turkey

## Abstract

The mol­ecule of the title compound, C_26_H_26_Br_4_O_6_, is located around a crystallographic inversion center. The dihedral angle between the central benzene ring and the outer benzene ring is 89.26 (1)°.

## Related literature

For information related to the synthesis of the title compound, see: Ford & Davidson (1993 ▶); Glombitza *et al.* (1985 ▶); Akbaba *et al.* (2010 ▶); Balaydın *et al.* (2009 ▶, 2010 ▶).
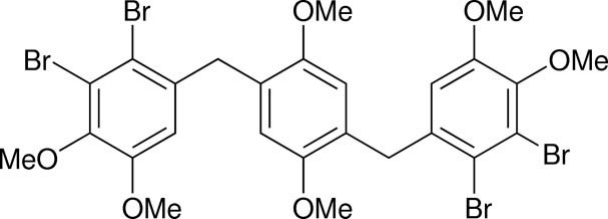


## Experimental

### 

#### Crystal data

C_26_H_26_Br_4_O_6_
*M* *_r_* = 754.07
Monoclinic, 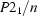

*a* = 11.193 (5) Å
*b* = 9.645 (4) Å
*c* = 13.212 (5) Å
β = 107.125 (5)°
*V* = 1363.1 (10) Å^3^
*Z* = 2
Mo *K*α radiation
μ = 5.94 mm^−1^
*T* = 293 K
0.3 × 0.2 × 0.1 mm

#### Data collection

Rigaku R-AXIS RAPID-S diffractometer
Absorption correction: multi-scan (Blessing, 1995 ▶) *T*_min_ = 0.250, *T*_max_ = 0.552
27932 measured reflections
2793 independent reflections
2564 reflections with *I* > 2σ(*I*)
*R*_int_ = 0.080

#### Refinement

*R*[*F* ^2^ > 2σ(*F* ^2^)] = 0.078
*wR*(*F* ^2^) = 0.125
*S* = 1.45
2793 reflections
166 parameters
H-atom parameters constrained
Δρ_max_ = 0.29 e Å^−3^
Δρ_min_ = −0.50 e Å^−3^

### 

Data collection: *CrystalClear* (Rigaku/MSC, 2005 ▶); cell refinement: *CrystalClear*; data reduction: *CrystalClear*; program(s) used to solve structure: *SHELXS97* (Sheldrick, 2008 ▶); program(s) used to refine structure: *SHELXL97* (Sheldrick, 2008 ▶); molecular graphics: *ORTEP-3 for Windows* (Farrugia, 1997 ▶); software used to prepare material for publication: *WinGX* (Farrugia, 1999 ▶).

## Supplementary Material

Crystal structure: contains datablocks global, I. DOI: 10.1107/S1600536810043758/gk2308sup1.cif

Structure factors: contains datablocks I. DOI: 10.1107/S1600536810043758/gk2308Isup2.hkl

Additional supplementary materials:  crystallographic information; 3D view; checkCIF report

## Figures and Tables

**Fig. 1.**
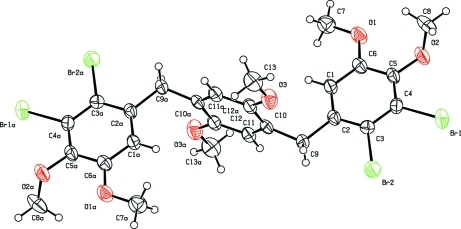
The molecular structure of the title compound with the atom-numbering scheme. Displacement ellipsoids are drawn at the 40% probability level. Atoms denoted as 'a' are generated by inversion operation 2-x,y,2-z.

## supplementary crystallographic information

### Comment

2,3-Dibromo-1-[4 -(2,3-dibromo-4,5-dimethoxybenzyl)-2,5-dimethoxybenzyl]-4, 5-dimethoxybenzene (1) was synthesized from reaction of bromoalcohol 2 (Ford & Davidson, 1993; Glombitza *et al.*, 1985) with 1,4-dimethoxybenzene in polyphosphoric acid (PPA) (Akbaba, *et al.* 2010; Balaydın *et al.*, 2010). From demethylation of this compound with BBr_3_, a bromophenol derivative can be easily obtained which may show important biological activities in further studies. The primary reason for the X-ray analysis of the title compound was to designate the position of Br atoms and methoxy groups and to obtain detailed information on the molecular structure. This information will be useful in structure-activity relationship (SAR) studies.

The title compound packs with half molecule in the asymmetric unit. Its molecular structure is shown in Fig. 1. Compound 1 includes three aromatic rings containing methoxy and bromide groups.

### Experimental

Polyphosphoric acid (PPA), prepared from conc. H_3_PO_4_ (85%, 0.57 g, 0.006 mmol) and P_2_O_5_ (1.03 g, 0.007 mmol), was heated to 80 °C in a beaker (100 ml) (Akbaba *et al.*, 2010; Balaydın *et al.*, 2010, 2009). To this mixture were added 1,4-dimethoxybenzene (0.138 g, 1 mmol) and synthesized bromoalcohol 2 (see Sscheme 2) (Ford & Davidson, 1993; Glombitza *et al.*, 1985) respectively and quickly. The mixture was stirred with a glass stick at 80 °C for 60 minutes and was cooled to room temperature. Mixture (50 ml) of water-ice and ethyl acetate (EtOA, 50 ml) was carefully added to the cooled mixture, respectively. The organic phase was separated and then the water phase was extracted with EtOAc (2x40 ml). The combined organic layers were dried over Na_2_SO_4_ and the solvent was evaporated. The product 1 (0.73 g, 97%) was obtained and crystallized from ethyl acetate/hexane as colorless crystals. *M*.p. 442–444 K. ^1^H-NMR (400 MHz, CDCl_3_) δ 6.71 (s, 2H), 6.67 (s, 2H), 4.09 (s, CH_2_, 4H), 3.83 (s, methoxide, 6H), 3.73 (s, methoxide,6*H*), 3.72 (s, methoxide, 6H); 13 C-NMR (100 MHz, CDCl_3_) δ 152.58 (C), 151.64 (C), 146.30 (C), 137.89 (C), 126.90 (C), 121.90(C), 118.01 (C), 114.01 (CH), 113.74 (CH), 60.71 (OCH_3_), 56.37 (OCH_3_), 56.32 (OCH_3_), 37.92 (CH_2_); IR (CH_2_Cl_2_, cm ^-1^): 2997,2935, 2829, 1546, 1507, 1463, 1421, 1402, 1371, 1310, 1282, 1212,1160, 1057, 1038,1006.

### Refinement

All H atoms were placed in calculated positions and allowed to ride on their carrier atoms with C—H = 0.93 - 0.97 Å and with *U*_iso_(H) = 1.2U_eq_(C) or *U*_iso_(H) = 1.5U_eq_(C_methyl_).

### Crystal data

**Table:**

C_26_H_26_Br_4_O_6_	*F*(000) = 740
*M**_r_* = 754.07	*D*_x_ = 1.837 Mg m^−^^3^
Monoclinic, *P*2_1_/*n*	Mo *K*α radiation, λ = 0.71073 Å
Hall symbol: -P 2yn	Cell parameters from 6447 reflections
*a* = 11.193 (5) Å	θ = 2.7–26.4°
*b* = 9.645 (4) Å	µ = 5.94 mm^−^^1^
*c* = 13.212 (5) Å	*T* = 293 K
β = 107.125 (5)°	Prism, colourless
*V* = 1363.1 (10) Å^3^	0.3 × 0.2 × 0.1 mm
*Z* = 2	

### Data collection

**Table:**

Rigaku R-AXIS RAPID-S diffractometer	2564 reflections with *I* > 2σ(*I*)
graphite	*R*_int_ = 0.080
ω scans	θ_max_ = 26.5°, θ_min_ = 2.7°
Absorption correction: multi-scan (Blessing, 1995)	*h* = −14→13
*T*_min_ = 0.250, *T*_max_ = 0.552	*k* = −12→12
27932 measured reflections	*l* = −16→16
2793 independent reflections	

### Refinement

**Table:**

Refinement on *F*^2^	Primary atom site location: structure-invariant direct methods
Least-squares matrix: full	Secondary atom site location: difference Fourier map
*R*[*F*^2^ > 2σ(*F*^2^)] = 0.078	Hydrogen site location: inferred from neighbouring sites
*wR*(*F*^2^) = 0.125	H-atom parameters constrained
*S* = 1.45	*w* = 1/[σ^2^(*F*_o_^2^) + (0.0035*P*)^2^ + 2.976*P*] where *P* = (*F*_o_^2^ + 2*F*_c_^2^)/3
2793 reflections	(Δ/σ)_max_ < 0.001
166 parameters	Δρ_max_ = 0.29 e Å^−^^3^
0 restraints	Δρ_min_ = −0.50 e Å^−^^3^

### Special details

**Table:**

Geometry. All s.u.'s (except the s.u. in the dihedral angle between two l.s. planes) are estimated using the full covariance matrix. The cell s.u.'s are taken into account individually in the estimation of s.u.'s in distances, angles and torsion angles; correlations between s.u.'s in cell parameters are only used when they are defined by crystal symmetry. An approximate (isotropic) treatment of cell s.u.'s is used for estimating s.u.'s involving l.s. planes.
Refinement. Refinement of *F*^2^ against ALL reflections. The weighted *R*-factor *wR* and goodness of fit *S* are based on *F*^2^, conventional *R*-factors *R* are based on *F*, with *F* set to zero for negative *F*^2^. The threshold expression of *F*^2^ > 2σ(*F*^2^) is used only for calculating *R*-factors(gt) *etc*. and is not relevant to the choice of reflections for refinement. *R*-factors based on *F*^2^ are statistically about twice as large as those based on *F*, and *R*- factors based on ALL data will be even larger.

### Fractional atomic coordinates and isotropic or equivalent isotropic displacement parameters (Å^2^)

**Table:**

	*x*	*y*	*z*	*U*_iso_*/*U*_eq_	
Br1	0.34470 (6)	0.34698 (9)	0.98736 (6)	0.0672 (3)	
Br2	0.53324 (6)	0.33264 (8)	0.83484 (5)	0.0593 (2)	
O1	0.6334 (4)	0.0000 (5)	1.2376 (3)	0.0564 (12)	
O2	0.4301 (4)	0.1588 (5)	1.1761 (3)	0.0560 (12)	
O3	1.0448 (4)	−0.2703 (5)	0.9540 (4)	0.0617 (13)	
C1	0.6977 (5)	0.0659 (6)	1.0834 (4)	0.0404 (13)	
H1	0.7682	0.0096	1.1038	0.049*	
C2	0.6735 (5)	0.1422 (6)	0.9902 (4)	0.0387 (13)	
C3	0.5684 (5)	0.2278 (6)	0.9617 (4)	0.0396 (13)	
C4	0.4892 (5)	0.2351 (6)	1.0257 (5)	0.0429 (14)	
C5	0.5125 (5)	0.1579 (6)	1.1164 (5)	0.0411 (14)	
C6	0.6183 (5)	0.0727 (6)	1.1462 (5)	0.0431 (14)	
C7	0.7351 (7)	−0.0943 (7)	1.2701 (6)	0.0602 (18)	
H7A	0.7309	−0.1595	1.2142	0.09*	
H7B	0.7307	−0.1431	1.3322	0.09*	
H7C	0.8124	−0.044	1.2858	0.09*	
C8	0.4678 (8)	0.2424 (9)	1.2687 (6)	0.076 (2)	
H8A	0.5509	0.2169	1.3096	0.114*	
H8B	0.4114	0.2284	1.3101	0.114*	
H8C	0.4667	0.3382	1.2487	0.114*	
C9	0.7554 (5)	0.1280 (7)	0.9173 (5)	0.0458 (15)	
H9A	0.7093	0.0755	0.8556	0.055*	
H9B	0.7691	0.22	0.8931	0.055*	
C10	0.8809 (5)	0.0600 (7)	0.9629 (4)	0.0430 (14)	
C11	0.9001 (6)	−0.0763 (6)	0.9392 (5)	0.0450 (14)	
H11	0.8332	−0.1287	0.8988	0.054*	
C12	1.0184 (5)	−0.1362 (6)	0.9752 (5)	0.0422 (14)	
C13	0.9451 (7)	−0.3596 (7)	0.9009 (6)	0.0652 (19)	
H13A	0.8909	−0.3738	0.9444	0.098*	
H13B	0.9784	−0.4471	0.8874	0.098*	
H13C	0.8986	−0.3182	0.835	0.098*	

### Atomic displacement parameters (Å^2^)

**Table:**

	*U*^11^	*U*^22^	*U*^33^	*U*^12^	*U*^13^	*U*^23^
Br1	0.0497 (4)	0.0838 (6)	0.0731 (5)	0.0230 (4)	0.0257 (4)	−0.0002 (4)
Br2	0.0595 (4)	0.0687 (5)	0.0533 (4)	0.0172 (3)	0.0221 (3)	0.0135 (3)
O1	0.063 (3)	0.062 (3)	0.055 (3)	0.009 (2)	0.035 (2)	0.015 (2)
O2	0.048 (3)	0.071 (3)	0.060 (3)	−0.007 (2)	0.034 (2)	−0.012 (2)
O3	0.056 (3)	0.055 (3)	0.078 (3)	0.005 (2)	0.025 (3)	−0.012 (2)
C1	0.040 (3)	0.043 (3)	0.044 (3)	0.005 (2)	0.020 (3)	0.000 (3)
C2	0.032 (3)	0.044 (3)	0.044 (3)	−0.006 (2)	0.017 (2)	−0.007 (3)
C3	0.039 (3)	0.044 (3)	0.036 (3)	0.000 (3)	0.010 (3)	−0.002 (3)
C4	0.032 (3)	0.050 (4)	0.048 (4)	0.004 (3)	0.012 (3)	−0.006 (3)
C5	0.033 (3)	0.049 (4)	0.047 (3)	−0.008 (3)	0.021 (3)	−0.012 (3)
C6	0.044 (3)	0.045 (3)	0.046 (3)	−0.006 (3)	0.022 (3)	−0.004 (3)
C7	0.070 (5)	0.055 (4)	0.060 (4)	0.004 (4)	0.025 (4)	0.004 (3)
C8	0.093 (6)	0.088 (6)	0.065 (5)	−0.004 (5)	0.053 (4)	−0.021 (4)
C9	0.036 (3)	0.065 (4)	0.042 (3)	0.007 (3)	0.020 (3)	0.007 (3)
C10	0.039 (3)	0.060 (4)	0.038 (3)	0.003 (3)	0.024 (3)	0.006 (3)
C11	0.042 (3)	0.055 (4)	0.043 (3)	0.000 (3)	0.020 (3)	−0.003 (3)
C12	0.045 (3)	0.045 (4)	0.043 (3)	0.007 (3)	0.023 (3)	0.002 (3)
C13	0.075 (5)	0.053 (4)	0.073 (5)	−0.001 (4)	0.031 (4)	−0.002 (4)

### Geometric parameters (Å, °)

**Table:**

Br2—C3	1.897 (6)	C9—H9B	0.97
Br1—C4	1.886 (6)	C11—H11	0.93
O1—C6	1.363 (6)	C8—H8A	0.96
O1—C7	1.422 (5)	C8—H8B	0.96
O2—C5	1.379 (6)	C8—H8C	0.96
O2—C8	1.421 (5)	C1—C6	1.385 (5)
O3—C12	1.374 (5)	C1—C2	1.390 (6)
O3—C13	1.420 (6)	C1—H1	0.93
C12—C10^i^	1.392 (6)	C6—C5	1.400 (6)
C12—C11	1.393 (6)	C4—C5	1.370 (6)
C3—C4	1.394 (6)	C13—H13A	0.96
C3—C2	1.395 (6)	C13—H13B	0.96
C10—C11	1.383 (6)	C13—H13C	0.96
C10—C12^i^	1.392 (6)	C7—H7A	0.96
C10—C9	1.507 (6)	C7—H7B	0.96
C9—C2	1.518 (5)	C7—H7C	0.96
C9—H9A	0.97		
			
C6—O1—C7	118.4 (5)	C6—C1—C2	120.9 (5)
C5—O2—C8	114.6 (5)	C6—C1—H1	119.6
C12—O3—C13	119.1 (5)	C2—C1—H1	119.6
O3—C12—C10^i^	115.5 (5)	C1—C2—C3	118.8 (5)
O3—C12—C11	124.1 (6)	C1—C2—C9	121.1 (5)
C10^i^—C12—C11	120.4 (5)	C3—C2—C9	120.0 (5)
C4—C3—C2	120.1 (5)	O1—C6—C1	125.0 (5)
C4—C3—Br2	120.3 (4)	O1—C6—C5	115.1 (5)
C2—C3—Br2	119.6 (4)	C1—C6—C5	119.9 (5)
C11—C10—C12^i^	118.8 (5)	C5—C4—C3	120.8 (5)
C11—C10—C9	120.8 (6)	C5—C4—Br1	118.2 (4)
C12^i^—C10—C9	120.2 (6)	C3—C4—Br1	120.9 (4)
C10—C9—C2	116.9 (5)	C4—C5—O2	120.7 (5)
C10—C9—H9A	108.1	C4—C5—C6	119.5 (5)
C2—C9—H9A	108.1	O2—C5—C6	119.7 (5)
C10—C9—H9B	108.1	O3—C13—H13A	109.5
C2—C9—H9B	108.1	O3—C13—H13B	109.5
H9A—C9—H9B	107.3	H13A—C13—H13B	109.5
C10—C11—C12	120.8 (6)	O3—C13—H13C	109.5
C10—C11—H11	119.6	H13A—C13—H13C	109.5
C12—C11—H11	119.6	H13B—C13—H13C	109.5
O2—C8—H8A	109.5	O1—C7—H7A	109.5
O2—C8—H8B	109.5	O1—C7—H7B	109.5
H8A—C8—H8B	109.5	H7A—C7—H7B	109.5
O2—C8—H8C	109.5	O1—C7—H7C	109.5
H8A—C8—H8C	109.5	H7A—C7—H7C	109.5
H8B—C8—H8C	109.5	H7B—C7—H7C	109.5
			
C13—O3—C12—C10^i^	173.5 (5)	C7—O1—C6—C5	176.8 (5)
C13—O3—C12—C11	−6.3 (9)	C2—C1—C6—O1	178.8 (5)
C11—C10—C9—C2	102.1 (7)	C2—C1—C6—C5	−0.5 (9)
C12^i^—C10—C9—C2	−81.7 (7)	C2—C3—C4—C5	−0.7 (9)
C12^i^—C10—C11—C12	−0.9 (9)	Br2—C3—C4—C5	178.8 (4)
C9—C10—C11—C12	175.4 (5)	C2—C3—C4—Br1	−178.7 (4)
O3—C12—C11—C10	−179.3 (5)	Br2—C3—C4—Br1	0.8 (7)
C10^i^—C12—C11—C10	0.9 (9)	C3—C4—C5—O2	−176.0 (5)
C6—C1—C2—C3	1.0 (8)	Br1—C4—C5—O2	2.0 (8)
C6—C1—C2—C9	−175.9 (5)	C3—C4—C5—C6	1.2 (9)
C4—C3—C2—C1	−0.4 (8)	Br1—C4—C5—C6	179.3 (4)
Br2—C3—C2—C1	−179.9 (4)	C8—O2—C5—C4	−102.1 (7)
C4—C3—C2—C9	176.5 (5)	C8—O2—C5—C6	80.7 (7)
Br2—C3—C2—C9	−3.0 (7)	O1—C6—C5—C4	180.0 (5)
C10—C9—C2—C1	−16.1 (8)	C1—C6—C5—C4	−0.7 (9)
C10—C9—C2—C3	167.1 (5)	O1—C6—C5—O2	−2.8 (8)
C7—O1—C6—C1	−2.6 (9)	C1—C6—C5—O2	176.6 (5)

Symmetry codes: (i) −*x*+2, −*y*, −*z*+2.
